# Placental endoplasmic reticulum stress in gestational diabetes: the potential for therapeutic intervention with chemical chaperones and antioxidants

**DOI:** 10.1007/s00125-016-4040-2

**Published:** 2016-07-12

**Authors:** Hong-wa Yung, Patji Alnæs-Katjavivi, Carolyn J. P. Jones, Tatiana El-Bacha, Michaela Golic, Anne-Cathrine Staff, Graham J. Burton

**Affiliations:** 1 Centre for Trophoblast Research, Department of Physiology, Development and Neuroscience, Physiological Laboratory, University of Cambridge, Downing Street, Cambridge, CB2 3EG UK; 2 Department of Obstetrics and Gynecology, Oslo University Hospital, Ullevål and Faculty of Medicine, University of Oslo, Oslo, Norway; 3 Centre for Maternal and Fetal Health, Institute of Human Development, University of Manchester, Manchester, UK; 4 Institute of Nutrition, Federal University of Rio de Janeiro, Rio de Janeiro, Brazil; 5 Experimental and Clinical Research Center, a cooperation between the Max Delbrück Center for Molecular Medicine in the Helmholtz Association and the Charité–Universitätsmedizin Berlin, Berlin, Germany; 6 Department of Obstetrics and Gynecology, Charité–Universitätsmedizin Berlin, Berlin, Germany; 7 Berlin Institute of Health (BIH), Berlin, Germany

**Keywords:** Antioxidants, Chaperones, Endoplasmic reticulum stress, Gestational diabetes, Metabolic acidosis, Placenta, Trophoblast, Unfolded protein response

## Abstract

**Aims/hypothesis:**

The aim of this work was to determine whether placental endoplasmic reticulum (ER) stress may contribute to the pathophysiology of gestational diabetes mellitus (GDM) and to test the efficacy of chemical chaperones and antioxidant vitamins in ameliorating that stress in a trophoblast-like cell line in vitro.

**Methods:**

Placental samples were obtained from women suffering from GDM and from normoglycaemic controls and were frozen immediately. Women with GDM had 2 h serum glucose levels > 9.0 mmol/l following a 75 g oral glucose tolerance test and were treated with diet and insulin when necessary. Western blotting was used to assess markers of ER stress. To test the effects of hyperglycaemia on the generation of ER stress, a new trophoblast-like cell line, BeWo-NG, was generated by culturing in a physiological glucose concentration of 5.5 mmol/l (over 20 passages) before challenging with 10 or 20 mmol/l glucose.

**Results:**

All GDM patients were well-controlled (HbA_1c_ 5.86 ± 0.55% or 40.64 ± 5.85 mmol/mol, *n* = 11). Low-grade ER stress was observed in the placental samples, with dilation of ER cisternae and increased phosphorylation of eukaryotic initiation factor 2 subunit α. Challenge of BeWo-NG with high glucose activated the same pathways, but this was as a result of acidosis of the culture medium rather than the glucose concentration per se. Addition of chemical chaperones 4-phenylbutyrate and tauroursodeoxycholic acid and vitamins C and E ameliorated the ER stress.

**Conclusions/interpretation:**

This is the first report of placental ER stress in GDM patients. Chemical chaperones and antioxidant vitamins represent potential therapeutic interventions for GDM.

**Electronic supplementary material:**

The online version of this article (doi:10.1007/s00125-016-4040-2) contains peer-reviewed but unedited supplementary material, which is available to authorised users.

## Introduction

Gestational diabetes mellitus (GDM) is a subtype of diabetes that arises de novo late in the second trimester or early in the third trimester of pregnancy. It represents a global health challenge, with incidences reaching 17.8% of total pregnancies [1]. Predisposing factors include maternal obesity, metabolic dysfunction and genetic susceptibility. Although the mechanisms are not fully understood, decreased insulin sensitivity and inadequate insulin secretion are two common causative factors. Women with GDM are at increased risk of developing other complications of pregnancy, including pregnancy-induced hypertension and pre-eclampsia [2]. They also have an increased lifetime risk for type 2 diabetes of approximately 30% [3]. For the fetus, GDM poses an increased risk of stillbirth, perinatal complications, macrosomia and growth restriction [4], and there is a greater predisposition to type 2 diabetes, obesity and metabolic and cardiovascular diseases later in life [5, 6].

Pregnancy is a pro-diabetogenic state due to changes in the endocrine regulation of maternal carbohydrate and lipid metabolism, which are essential for elevating circulating blood glucose to meet fetal demands. Consequently, maternal organs and peripheral tissues are adapted for glucose intolerance and insulin resistance. As pregnancy advances, the placenta is the major driver of these maternal metabolic adaptations [7]. Although widely recognised as an organ of nutrient and gaseous exchange, the placenta secretes an array of polypeptide hormones, growth factors, cytokines and adipokines that modulate maternal metabolism and fetal growth. Human placental lactogen (hPL) and prolactin induce beta cell expansion and insulin release in the maternal pancreas [8], while human placental growth hormone (hPGH) and adipokines cause peripheral insulin resistance [9]. Maternal serum concentrations of hPL and hPGH increase up to 30- and eightfold, respectively, during pregnancy [10]. The placenta also secretes leptin, regulated by a placenta-specific upstream enhancer [11], and adiponectin [12] for regulation of maternal lipid metabolism, favouring fatty acids as a fuel source in maternal tissues. In normal pregnancy, these factors act in concert to redistribute maternal energy resources, increasing their availability for the fetus. However, perturbation of placental function potentially disrupts their bioactivity, altering the normal balance of maternal metabolism and resulting in GDM.

Organs with high polypeptide endocrine activity, such as the pancreas and placenta, are susceptible to endoplasmic reticulum (ER) stress. The ER coordinates the biosynthesis and post-translational modification of secreted proteins and membrane receptors, as well as maintaining calcium homoeostasis in cells. Therefore, any perturbation of the ER intraluminal environment can severely affect the bioactivity of secreted proteins. Accumulation of misfolded proteins in the ER provokes activation of an integrated network of signalling pathways known as the unfolded protein response (UPR). These pathways aim to restore ER homeostasis and involve protein kinase RNA-like ER kinase (PERK)–eukaryotic initiation factor 2 subunit α (eIF2α), which attenuates non-essential protein synthesis, and ATF6 (activating transcription factor 6) and inositol-requiring enzyme 1 (IRE1)–X-box binding protein 1 (XBP-1), which promote the synthesis of ER-resident chaperones to increase folding capacity. If these pathways fail to restore homeostasis, apoptosis is activated to eliminate the damaged cells [13].

Research on the role of ER stress in human diabetes has advanced rapidly and targeting to ameliorate ER stress has become a therapeutic intervention [14]. In type 2 diabetes, ER stress-induced dysfunction of pancreatic beta cells reduces insulin production as well as its bioactivity, resulting in hyperglycaemia [15]. The current view is that GDM is a pre-diabetic state and, although there may be several underlying causes, the majority of cases appear to be due to pre-existing low-grade chronic beta cell dysfunction [16]. Consequently, insufficient insulin is secreted to overcome the pregnancy-induced insulin resistance in the maternal organs/peripheral tissues [17].

We have demonstrated the central role of placental ER stress in the pathophysiology of pregnancy complications, including fetal growth restriction and early-onset pre-eclampsia [18, 19]. In this study, we first investigate evidence of ER stress in placentas from women with GDM. We then use an in vitro trophoblastic-like cell model to elucidate the mechanisms by which hyperglycaemia may induce placental ER stress. Finally, we examine the ability of chemical chaperones and antioxidants to mitigate ER stress in trophoblast-like cells under high glucose concentrations.

## Methods

### Materials

All chemicals were purchased from either Sigma-Aldrich (Dorset, UK) or Fisher Scientific UK (Loughborough, UK) except where otherwise stated. Antibodies against p-eIF2α (Ser51), eIF2α, calreticulin and protein disulphide isomerase (PDI) were from Cell Signalling Technology (NEB, Hitchin, UK). Antibodies against XBP-1 and glucose related protein (GRP) 94 were from Abcam (Cambridge, UK), anti-Desmoplakin 1/2 was from AbD Serotec (Oxford, UK), anti-GRP78 was from Transduction Laboratories (BD Biosciences, Oxford, UK), and anti-β-actin was from Sigma-Aldrich. All antibodies were used according to manufacturer’s instruction except where otherwise stated.

### Participant selection and placental tissue collection

Placental samples were obtained from Oslo Pregnancy Biobank at Oslo University Hospital, Norway (www.oslo-universitetssykehus.no/opb). Twenty-one placentas from singleton pregnancies were delivered by non-laboured elective Caesarean section. The study was approved by the South-Eastern Norway Regional Committee for Medical and Health Research Ethics and informed consent was obtained from all participants.

Pregnant women with (*n* = 11) and without (*n* = 10) GDM were originally recruited onto the study. GDM participants had 2 h serum glucose levels > 9.0 mmol/l following a 75 g oral glucose tolerance test. GDM participants were treated by diet, and insulin was started if necessary, based on clinical indication. The control group (‘Control 1’) was free from pre- and post-gestational diseases (including chronic diseases and pregnancy complications), and had healthy pregnancy outcomes. Thirty per cent of individuals in Control 1 had a pre-gestational BMI >25. A second control group (‘Control 2’; *n* = 8) was also recruited for comparison with normoglycaemic individuals with overweight/obesity (*n* = 7). All participants in Control 2 had a pre-gestational BMI <25 and, as with Control 1, were free from pre- and post-gestational diseases and had healthy pregnancy outcomes.

HbA_1c_ measurements were performed at standardised intervals during the participant’s pregnancy, in keeping with the institution’s guidelines for antenatal follow-up of diabetes in pregnancy. Capillary samples were collected from participants using Accu-chek Safe-T-pro Plus lancets (Roche Diagnostics, Indianapolis, IN, USA) and added to a reagent kit for whole blood (DCA Systems Hemoglobin A_1c_ Reagent Kit, Siemens Healthcare Diagnostics, Tarrytown, NY, USA). The HbA_1c_ concentration was analysed in the clinic (‘at point of care’) using the Siemens/Bayer DCA 2000+ Analyzer from 2001–2013 (replaced by Siemens DCA Vantage Analyzer as of 2014 [Global Division, Siemens Healthcare Diagnostics, Tarrytown, NY, USA; see www.siemens.com/diagnostics]). To convert values for HbA_1c_ in % (National Glycohemoglobin Standardization Program [NGSP] units) into mmol/mol (International Federation of Clinical Chemistry and Laboratory Medicine [IFCC] units), subtract 2.15 and multiply by 10.929.

In all participants, gestational age was determined during pregnancy by ultrasound scanning between 17 and 20 weeks of gestation and all participants delivered at the same gestational stage. Placental samples were processed immediately following delivery. Biopsies were selected from a macroscopically normal-looking, centrally located lobule, avoiding the decidual and membrane layers. Samples were snap-frozen in liquid nitrogen and stored at −80°C. All samples were analysed on a non-blinded basis.

### Generation of a new line of BeWo cells, BeWo-NG

BeWo cells were a gift from Professor Charnock-Jones (Department of Obstetrics and Gynaecology, University of Cambridge, UK) and were verified using a panel of markers for trophoblast cells [20]. They were not tested for *Mycoplasma* contamination. BeWo cells are routinely cultured in medium with a high concentration of glucose, such as DMEM/F12 (containing 17.1 mmol/l glucose). This non-physiological hyperglycaemic environment renders these cells inappropriate for any glucose-related study. Therefore, a new line, BeWo-NG, was generated by culturing cells in the physiological glucose concentration of 5.5 mmol/l in modified DMEM/F12 medium, which was prepared by adding 0.99 g of glucose into 1 l of glucose-free DMEM/F12 medium (US Biological, Salem, MA, USA). Details are described in the ESM Methods; ‘Generation of new line of BeWo cells, BeWo-NG and culturing conditions’. After 20 passages, the BeWo-NG cells showed no difference in proliferation rate compared with parental BeWo cells (ESM Fig. 1a). Treatment with 10 μmol/l forskolin A induced syncytialisation as indicated by loss of Desmoplakin staining using anti-human Desmoplakin 1/2 antibody (AbD Serotec, Oxford, UK) and production of human chorionic gonadotrophin (ESM Fig. 1b, c). Details of Desmoplakin 1/2 immunocytochemical staining are described in ESM. These results confirmed that the BeWo-NG cells retained properties similar to those of the parental BeWo cells.

### Western blot analysis

The levels of total and phosphorylated proteins and kinases were measured by western blotting as described in the ESM Methods. All antibodies were used at a 1:1000 dilution except GRP78 and β-actin, which were used at a 1:10,000 dilution. All primary antibodies were diluted in Tris-buffered saline (TBS) containing 0.1% (vol./vol.) Tween 20 and incubated overnight at 4°C, followed by a few hours at room temperature. The antibodies for ER and oxidative stress markers were validated with cell lysates prepared from the cells treated with either ER stress inducers (tunicamycin [Sigma-Aldrich] and thapsigargin [Sigma-Aldrich]) or exposure to repetitive hypoxia-reoxygenation, as shown in previous publications [18, 24].

### Measurement of pH

The pH of media was determined using a pH meter (JENCO model 60, Lazar Research Laboratories, Los Angeles, CA, USA) connected to a micro pH electrode (Lazar Research Laboratories). Each experimental condition was performed in duplicate.

### Lactate assay

Lactate accumulation in the culture media was evaluated enzymatically in hydrazine/glycine buffer (pH 9.2), containing 5 mg/ml β-NAD^+^ and 30 U/ml lactate dehydrogenase to a final volume of 1 ml [21]. The reaction was started by addition of a sample. The change in absorbance because of NADH formation was monitored for 4 min at 340 nm using a Helios α UV-VIS spectrophotometer (Unicam, Cambridge, UK). A lactate calibration curve ranging from 10 to 150 μmol/l was constructed and used to calculate lactate concentrations in samples.

### Electron microscopy

Samples from normal and GDM placentas were fixed immediately after delivery in 2.5% glutaraldehyde (wt/vol.) in 0.1 mol/l cacodylate buffer for 4 h, post-fixed in 1% osmium and then embedded in Araldite resin. Thin sections were stained with lead citrate and uranyl acetate, and viewed using a Philips CM100 microscope (FEI, Hillsboro, OR, USA). All chemicals were purchased from TAAB Laboratories Equipment (Berks, UK)

### Statistical analysis

Statistical analyses were performed in GraphPad Prism 6.0 (GraphPad Software, La Jolla, CA, USA). Differences were tested using the non-parametric Mann–Whitney *U* test or parametric Student’s *t* test, with *p* < 0.05 being considered significant. For multiple comparisons, differences were tested using non-parametric Kruskal–Wallis test, followed by Dunn’s multiple comparisons test.

## Results

The GDM and control groups were similar regarding gestational age at delivery and maternal age (Table 1). Pre-pregnancy and delivery maternal BMI, were significantly higher in the GDM group than in the control group, whereas birthweights and placental weights were not significantly different. All GDM patients showed elevated levels of HbA_1c_ (5.86 ± 0.55% or 40.64 ± 5.85 mmol/mol, *n* = 11), indicating chronic hyperglycaemia. Although the HbA_1c_ level was not measured in the control group, a previous study reported a value of approximately 5% or 31 mmol/mol in the third trimester of normal pregnancy [22].

**Table 1:** Clinical characteristics of the participants according to study group

Characteristic	Control 1 (*n* = 10)	GDM (*n* = 11)	Control 2 (*n* = 8)	Non-GDM overweight/obese (*n* = 7)	*p* value (ANOVA)
Maternal age (years)	32 ± 1.5	34.3 ± 1.4	33.5 ± 2.4	33.7 ± 0.5	NS
Gestational age (weeks)	38.1 ± 0.2	38.9 ± 0.4	38.8 ± 0.2	39 ± 0.2^b^	0.013
Birthweight (g)	3582 ± 104	4195 ± 239	3595 ± 136	3830 ± 181	NS
Placental weight (g)	647 ± 52	767 ± 74	661 ± 62	680 ± 109	NS
Pre-pregnancy BMI (kg/m^2^)	22.8 ± 1.2	30.6 ± 1.3^a,^	21 ± 0.2	29 ± 1.0^b^	0.0004
Rate of pre-pregnancy overweight/obesity (%)					
BMI ≥ 25 kg/m^2^	30	72.7	0	100	
BMI ≥ 30 kg/m^2^	0	36.3	0	28.6	
Delivery BMI (kg/m^2^)	29.1 ± 1.2	34.7 ± 0.9^b^	27.3 ± 1.1	33.1 ± 1.2	0.0065
Capillary HbA_1c_ (late third trimester)					
(%)	5^c^	5.86 ± 0.55	5^c^	–	
(mmol/mol)	31^c^	40.64 ± 5.85	31^c^		
Intervention during GDM pregnancy (*n)*					
Insulin + diet	–	5	–	–	
Diet	–	6	–	–	

Values are expressed as mean ± SEM, unless stated otherwise

The ‘Control 1’ cohort is the initial normoglycaemic control group, containing 30% overweight/obese women with a pre-pregnancy BMI > 25. ‘Control 2’ is a second normoglycaemic control group of non-obese women with a pre-pregnancy BMI < 25

‘Overweight/obese GDM’ comprise of a subset of ‘GDM’ participants

Differences were tested using the non-parametric Kruskal–Wallis test, followed by Dunn’s multiple comparisons test

^a^Statistically significant compared with Control 1

^b^Statistically significant compared with Control 2

^c^HbA_1c_ was not measured in normal pregnancies; reference values during the third trimester were those measured by Neilsen et al [21]

### Low-grade ER stress is increased in GDM placentas

**Fig. 1:**
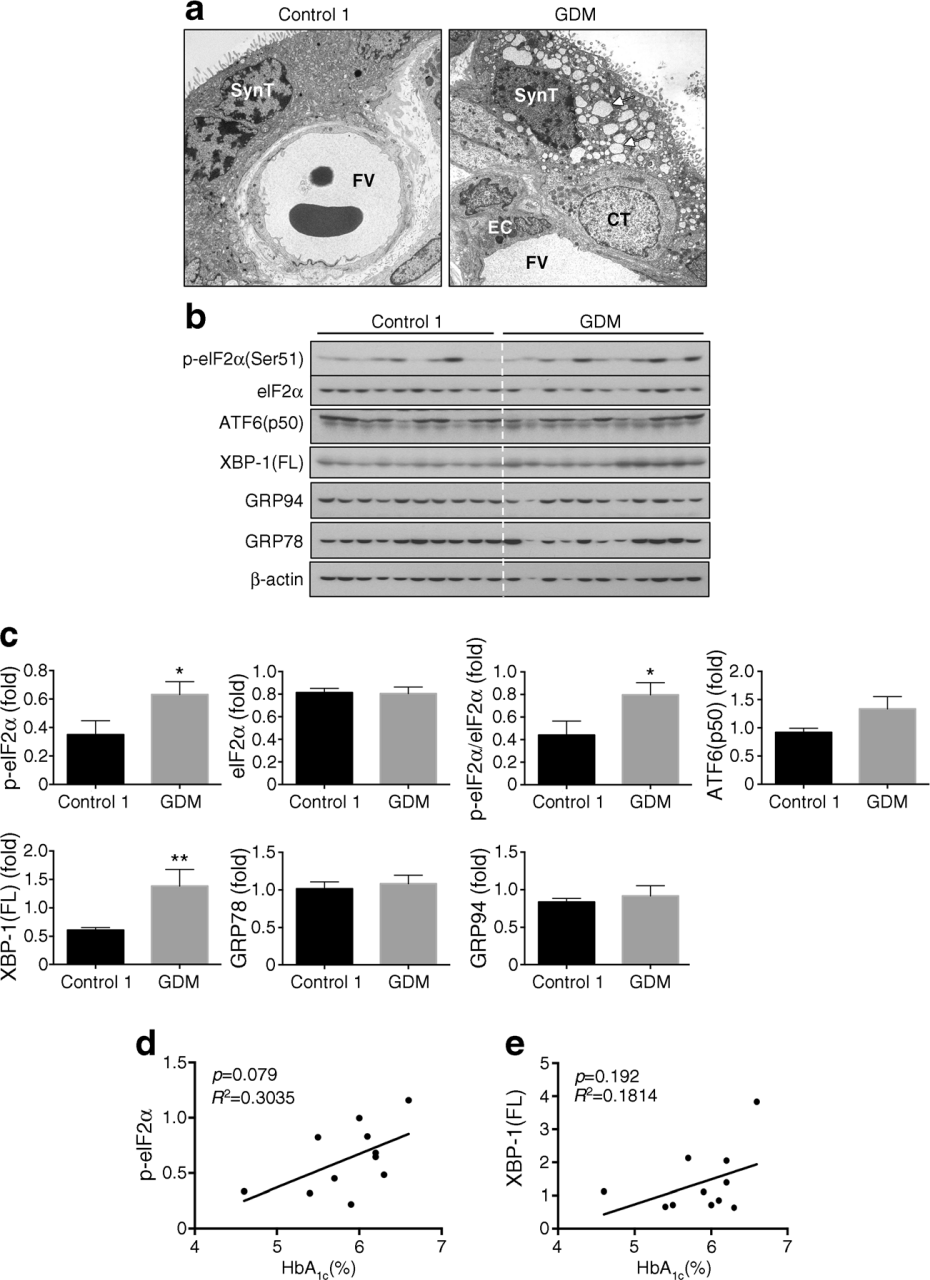
Low-grade ER stress in the GDM placentas. (**a**) Electron micrographs showing dilated ER cisternae (arrows) in the syncytiotrophoblast (SynT) but not in the cytotrophoblast (CT) or endothelial cells (EC). FV, fetal vessel. Magnification ×5000. (**b**) Representative blots of p-eIF2α, eIF2α, ATF6α, unspliced XBP-1 (XBP-1(FL)), GRP94 and GRP78. β-actin was used as loading control. (**c**) Quantification of the band intensity from (**b**). The *y*-axis shows the relative level of phosphorylated or total protein. Phosphorylation status of eIF2α is presented as the ratio between phosphorylated and total protein. All protein levels are normalised to β-actin. Data are presented as mean ± SEM, *n* = 10 or 11; *p < 0.05; **p < 0.01 vs control. (**d**, **e**) Correlation between HbA_1c_ and (**d**) p-eIF2α and (**e**) XBP-1(FL). In (**d**, **e**), the concentration of HbA_1c_ was plotted against relative ratio of p-eIF2α or XBP-1(FL) and a linear regression line added

Electron microscopy revealed mild to moderate dilatation of the ER cisternae, a hallmark of ER stress, in the syncytiotrophoblast of placentas from GDM pregnancies compared with normoglycaemic controls (Fig. 1a). The normal ER cisternae and mitochondrial cristae in the cytotrophoblast and endothelial cells confirmed that these appearances were not fixation artefacts (Fig. 1a).

To assess the severity of ER stress in the GDM placentas, the activity of the PERK, ATF6 and IRE1 pathways was monitored by examining their corresponding downstream effectors. Results revealed an increase of >8 in phosphorylation of eIF2α (p-eIF2α; Fig. 1 b, c), but no evidence of splicing of *XBP-1*. There was, however, a ∼1.2-fold elevation of the full-length XBP-1(FL) protein translated from the unspliced *XBP-1* mRNA. The correlation between levels of p-eIF2α and HbA_1c_ did not reach statistical significance (p-eIF2α, *p* = 0.079, Fig. 1d). ATF6 is processed by specific proteases to generate a potent ATF6(p50) transcription factor [16]. Although the ATF6α antibody used recognises both the full-length ATF6α(p90) and the active form (ATF6α[p50]) only the active form was detected (Fig. 1c). The level of ATF6α(p50) in the GDM placentas showed no significant difference (*p* = 0.09) compared with control placentas. Furthermore, the downstream targets of ATF6α(p50), GRP78 and GRP94, were unchanged compared with the controls. To summarise, both ultrastructural analysis and ER molecular markers demonstrate the existence of mild ER stress in placentas from GDM pregnancies.

Women with GDM had a pre-pregnancy BMI significantly higher than that of women in the control group, Control 1 (Table 1). Therefore, we investigated the contribution of obesity in the activation of placental ER stress. To allow for comparison with a solely non-obese control group, a second control group comprising placentas from non-obese women (BMI <25) with normal pregnancies (Control 2) was also collected. The pre-pregnancy BMI of the overweight/obese women was significantly higher than that of Control 2 (29 ± 1 vs 21 ± 0.2 kg/m^2^, *p* = 0.0004) and was comparable with that of all GDM cases (30.6 ± 1.3 kg/m^2^; Table 1). Immunoblotting revealed there to be no significant change in ER stress markers p-eIF2α and GRP78 in the overweight/obese women (ESM Fig. 2a, b). However, the expression of XBP-1, which was increased in the GDM placentas, was significantly reduced (ESM Fig. 2b). Additionally, there was no correlation between the p-eIF2α level in the placentas and maternal BMI in GDM (ESM Fig. 2c). To conclude, these results exclude the possibility of the increased ER stress in GDM placenta arising from maternal obesity.

### High glucose does not directly activate ER stress in trophoblast-like cells

To elucidate whether the placental ER stress observed in GDM pregnancies is the result of maternal hyperglycaemia, an in vitro model using trophoblast-like cells treated with high concentrations of glucose was introduced. As ER stress was observed solely in the syncytiotrophoblast, BeWo-NG cells were treated with forskolin A to induce differentiation into syncytial masses prior to experimentation.

**Fig. 2:**
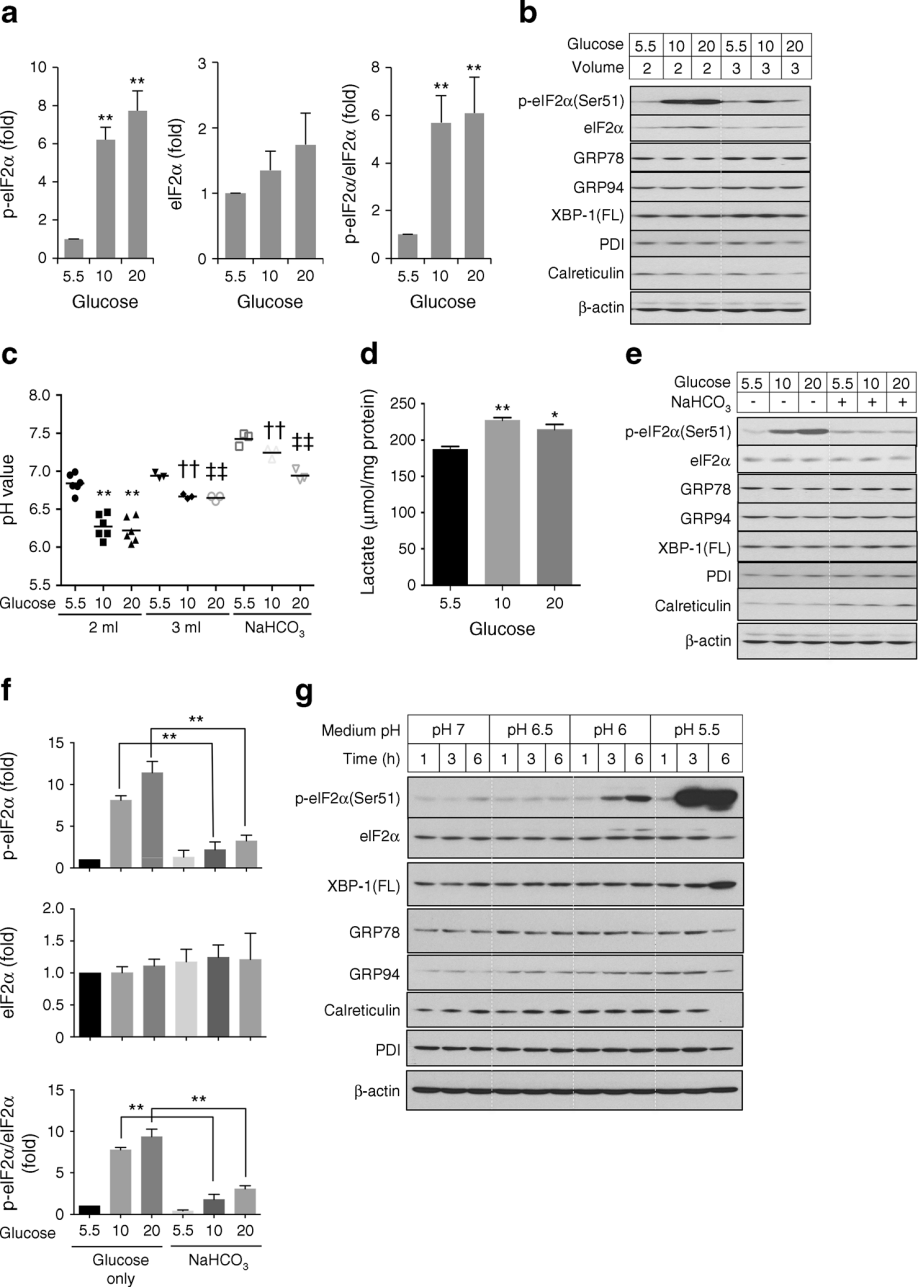
ER stress induced by high glucose is mediated by metabolic acidosis. BeWo-NG cells were cultured in serum-free media containing 5.5, 10 and 20 mmol/l glucose in combination with various experimental conditions for 24 h. ER stress markers were measured by western blotting, with β-actin or Ponceau S staining as loading control. (**a**) Phosphorylation of eIF2α under different glucose concentrations (mmol/l). The *y*-axis shows the relative level of p-eIF2α, eIF2α and the ratio of p-eIF2α/eIF2α. **p < 0.01 vs 5.5 mmol/l glucose. (**b**) Representative blots showing effect of changing medium volume on phosphorylation of eIF2α. Cells were treated with different glucose concentrations (mmol/l) in 2 ml (control) or 3 ml incubation volume. (**c**) Effect of changing medium volume and buffering capacity on pH induced by high glucose (10 or 20 mmol/l). **p < 0.01 vs 5.5 mmol/l glucose within the 2 ml glucose-only control group; ^††^ *p* < 0.01 vs 10 mmol/l glucose in the 2 ml glucose-only control group; ^‡‡^ *p* < 0.01 vs 20 mmol/l glucose in the 2 ml glucose-only control group. (**d**) Lactate production in high glucose medium. *p < 0.05 and **p < 0.01 vs 5.5 mmol/l glucose. (**e**) Representative western blots showing phosphorylation of eIF2α in relation to medium buffering capacity. Cells were cultured with different glucose concentrations (mmol/l) in the presence or absence of additional NaHCO_3_ (15 mmol/l) for 24 h. (**f**) Quantification of p-eIF2α with or without NaHCO_3_. The *y*-axis shows the relative level of p-eIF2α and eIF2α and the ratio of p-eIF2α/eIF2α. **p < 0.01 for presence vs absence of NaHCO_3_ at the same glucose concentration. (**g**) Effect of acidified culture media on induction of ER stress. The pH of the culture medium containing 5.5 mmol/l glucose was adjusted to 7, 6.5, 6 and 5.5, and the BeWo-NG cells were exposed to these media for 1, 3 or 6 h. Data are presented as mean ± SEM or as immunoblot images, *n* = 3–6

BeWo-NG cells adapted to a 5.5 mmol/l physiological glucose concentration were challenged with 10 and 20 mmol/l glucose for 24 h. The high glucose treatments induced an over sixfold elevation of p-eIF2α (Fig. 2a). However, other ER stress markers, GRP78, GRP94 and XBP-1(FL), and the ER residential proteins protein disulfide isomerase (PDI) and calreticulin, appeared to be unchanged (Fig. 2b).

A change of the pH indicator (Phenol Red) in the modified DMEM/F12 medium suggested that the high glucose concentrations caused metabolic acidosis. Indeed, while the mean pH of the 5.5 mmol/l glucose medium dropped from 7.0 to 6.8 after 24 h, in both the 10 and 20 mmol/l glucose media it was reduced to ∼6.2 (Fig. 2c). These reductions in pH were partially a result of lactate accumulation as there was a ∼23% and ∼15% increase in lactate in the media containing 10 and 20 mmol/l glucose, respectively (Fig. 2d).

To determine whether metabolic acidosis, per se, can induce ER stress, the volume of the medium was increased from 2 to 3 ml to dilute the metabolites. As a consequence, the pH of the media containing 10 and 20 mmol/l glucose was partially restored to ∼6.6 (Fig. 2c). Phosphorylation of eIF2α was greatly decreased (Fig. 2b), while levels of all other ER stress markers and residential proteins remained unchanged. To summarise, these results suggest that high glucose is unlikely to be a direct inducer of ER stress.

### High glucose-induced acidosis triggers mild ER stress in trophoblast-like cells

To further explore the potential role of metabolic acidosis in activation of ER stress in BeWo-NG cells, two approaches were introduced. First, an extra 15 mmol/l of sodium bicarbonate (NaHCO_3_) was added to the culture medium, raising the concentration from 14 to 29 mmol/l, to increase its buffering capacity. This caused the basal pH value to rise to ∼7.5 (Fig. 2c). After incubation for 24 h the pH of all the media with added NaHCO_3_ were significantly higher than the pH of media without the added NaHCO_3_ (Fig. 2c). In the 10 and 20 mmol/l glucose media, the values were ∼7.0. Restoration of the pH reduced p-eIF2α by ∼threefold (Fig. 2f), while levels of other ER stress markers and residential proteins appeared to remain constant (Fig. 2e).

Next, BeWo-NG cells were exposed for 1–6 h to media containing 5.5 mmol/l glucose with different acidities, ranging from pH 5.5 to 7.0. Culture for up to 6 h did not alter the pH values in the media, eliminating the possibility of any effect from accumulating metabolites (data not shown). Media with a pH higher than 6.5 did not induce ER stress (Fig. 2g). There was a time-dependent increase in p-eIF2α in medium with a pH of 6.0, but other ER stress markers remained constant. In medium of pH 5.5, p-eIF2α was increased strongly after only 3 h, and the level of XBP-1(FL) rose after 6 h (Fig. 2g). Phase-contrast microscopy examination revealed condensed nuclei and loss of membrane integrity; potential signs of necrosis (data not shown). A marked reduction of the ER residential proteins PDI and calreticulin (Fig. 2g), and of the cytosolic protein p38 kinase (ESM Fig. 3a), confirmed that the cells were losing both ER and plasma membrane integrity under these conditions.

To summarise, these findings suggest that exposure to a low pH can alter ER homeostasis, thereby activating the UPR.

### ER molecular chaperones and antioxidants suppress high glucose-induced ER stresses

**Fig. 3:**
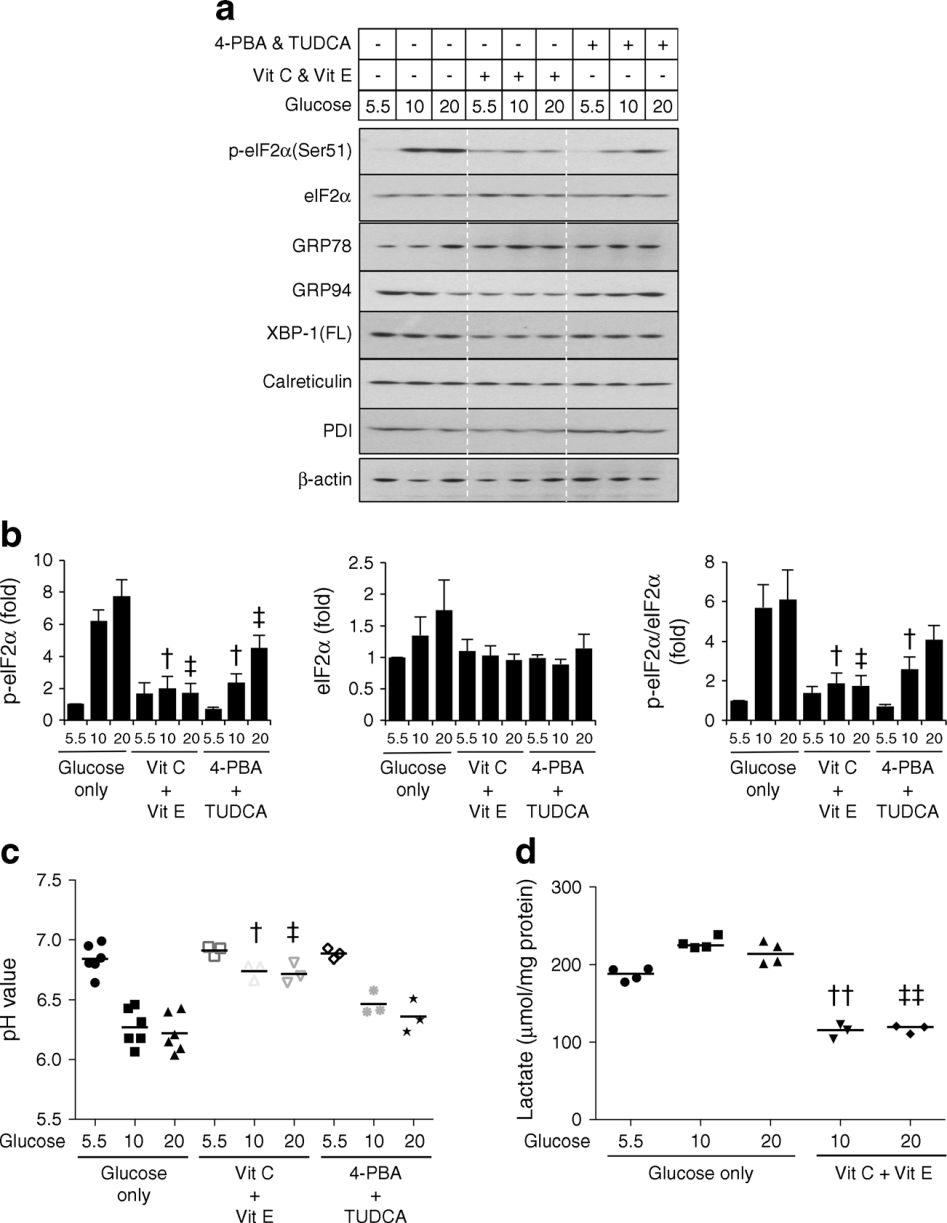
Both chemical chaperones and antioxidants effectively suppress high glucose-induced ER stress, but only antioxidants reduce the degree of metabolic acidosis. BeWo-NG cells were treated with different concentrations of glucose in the presence of either chemical chaperones, 4-PBA (500 μmol/l) and TUDCA (250 μmol/l), or vitamin C (Vit C; 500 μmol/l) or vitamin E (Vit E; 500 μmol/l) for 24 h. (**a**) Levels of ER stress markers were determined by western blotting. β-actin was used as loading control. (**b**) Phosphorylation status is presented as the ratio between phosphorylated and total protein. Data are presented as mean ± SEM, *n* = 3. The *y*-axis shows the relative level of p-eIF2α eIF2α or the ratio of p-eIF2α/eIF2α. (**c**) pH of the culture media after the experiment. (**d**) Effect of vitamins on high glucose-induced lactate production. Data are presented as median, *n* = 3–6. ^†^ *p* < 0.05 and ^††^ *p* < 0.01 vs 10 mmol/l glucose in the glucose-only control group; ^‡^ *p* < 0.05 and ^‡‡^ *p* < 0.01 vs 20 mmol/l glucose in the glucose-only control group

Chemical chaperones, including 4-phenylbutyrate (4-PBA) and tauroursodeoxycholic acid (TUDCA), have been widely demonstrated to be effective in reducing ER stress [23], while antioxidants, such as vitamins C and E, suppress oxidative stress in placental explants [24]. Therefore, we investigated the effects of chemical chaperones and antioxidants in the suppression of high glucose-induced ER stress. Both chemical chaperones and antioxidants were effective in reducing phosphorylation of eIF2α (Fig. 3a, b), while all other ER stress markers and residential proteins remained unchanged (Fig. 3a). Surprisingly, vitamins, but not chemical chaperones, significantly restored the pH of the normal media to ∼6.7 and reduced lactate accumulation by over 50% in the presence of 10 and 20 mmol/l glucose (Fig. 3c, d). Vitamin C or vitamin E alone suppressed high glucose-induced ER stress and restored the pH, but were not as effective when combined (data not shown). These results reveal potential beneficial effects of both chemical chaperones and vitamins C and E in reducing ER stress. Additionally, not only can the vitamins suppress the ER stress induced by high glucose but they may also be used to prevent hyperglycaemia-induced acidosis.

## Discussion

In this study, we provide the first evidence for the existence of placental ER stress in women with established GDM, as demonstrated by dilatation of ER cisternae in the syncytiotrophoblast and increased p-eIF2α and unspliced XBP-1 protein. Evidence for activation of the IRE1α arm remains elusive because of inconsistent signals from the downstream effectors, with no splicing of XBP-1, but potential activation of the TNF receptor-associated factor 2 (TRAF2)–c-Jun N-terminal kinase (JNK) pathway (ESM Fig. 4). To conclude, the results suggest that placental ER stress in GDM is mild compared with that observed in pregnancies complicated by fetal growth restriction and early-onset pre-eclampsia [18, 25]. It must be remembered, however, that the GDM placentas studied came from women whose glucose levels were well-controlled, as evidenced by mean HbA_1c_ levels which were only just outside the normal range. The severity of placental pathology in individuals with diabetes is generally related to the quality of glucose control [26].

To test whether elevated glucose concentrations per se are able to induce ER stress, we developed a new trophoblast-like cell line adapted to physiological glucose concentrations, BeWo-NG. Use of these cells revealed that high glucose concentrations induce ER stress in vitro, most likely through metabolic acidosis. Activation of the UPR was limited to the PERK signalling arm. Severe acidosis induces ER stress in other cell types [27]. Although the mechanisms are elusive, the activity of Ca^2+^-ATPases is susceptible to acidosis and there is a pH-dependent Ca^2+^ efflux from the intracellular storage compartments. Acidification of the medium (from pH 7.0 to 6.5) causes release of Ca^2+^ from the ER and loss of ER homeostasis [28], activating the UPR. Lactic acid has been demonstrated to induce phosphorylation of eIF2α in cancer cells, possibly through this mechanism [29]. In GDM, excessive maternal blood glucose could facilitate overproduction of lactate in the placenta, resulting in metabolic acidosis. Unlike many organs, in which lactate production is usually under hypoxic conditions, the placenta synthesises considerable quantities of lactate under aerobic conditions [30].

The human placenta expresses all five isozymes of lactate dehydrogenase, which catalyses the inter-conversion of lactate and pyruvate in the glycolytic pathway [31]. The activity of only one of the five isozymes is increased in trophoblast cells upon hypoxia [30], suggesting that the majority of the placental isozymes may facilitate lactate production aerobically. The amount of lactate produced by the placenta is directly proportional to the maternal glucose concentration, as elevation of the glucose concentration from 4.2 to 10.9 mmol/l during placental perfusion in vitro induced a fourfold increase in lactate production. However, raising the glucose concentration further had no additional effect [32], consistent with our observation of no change in the pH value of media containing 10 or 20 mmol/l glucose. Unfortunately, data on placental lactate production in vivo in GDM patients are not available. A study by Taricco et al, using indirect measures, reported a 23% increase of lactate concentration in the umbilical vein in GDM pregnancies [33]. Hence, the placenta is likely producing more lactate in GDM and transporting it to the fetus for metabolism.

An alternative explanation is that the placental ER stress reflects the maternal pro-inflammatory environment associated with obesity [34], a potentially confounding variable as our participants were not matched for pre-pregnancy BMI. However, placentas from normoglycaemic obese women did not show ER stress. Furthermore, adding palmitic acid to BeWo-NG cells during a high glucose challenge did not exacerbate the ER stress (ESM Fig. 2a, b, d), indicating that high levels of fatty acids are unlikely to be a causative factor. Finally, there was no correlation between the level of p-eIF2α and the BMI of the women at the time of delivery (ESM Fig. 2c). Together, these results suggest that obesity is unlikely to be the cause of the placental ER stress observed in GDM.

Oxidative stress is also a strong inducer of ER stress in the trophoblast [35]. However, the existence of oxidative stress in GDM placentas remains contentious as both increased and unchanged stress have been reported [36, 37]. This may be due to differences in the percentages of women under dietary control (85% vs 53%) and insulin therapy (15% vs 47%). Nevertheless, the GDM placentas used in this study (54% of which were from those under dietary control) showed only a mild level of oxidative stress as indicated by activation of JNKs without significant changes in p38 kinase phosphorylation and heat shock proteins (ESM Fig. 4). We speculate that placental ER and oxidative stress may occur to a greater extent in GDM pregnancies with more severe metabolic acidosis. Indeed, treating BeWo-NG cells with low-pH media strongly activated the oxidative stress marker p38 kinase phosphorylation (ESM Fig. 3a), similar to treatment with high glucose concentrations (ESM Fig. 3b). These results suggest that the oxidative stress observed in GDM placentas may be triggered at least in part by placental metabolic acidosis. Further studies are required to confirm this hypothesis.

Finally, we demonstrated the potential use of chemical ER chaperones and antioxidant vitamins as therapeutic interventions to prevent glucose-induced placental ER stress. Chemical chaperones had a similar efficacy to that of antioxidants in the suppression of ER stress at a glucose concentration of 10 mmol/l, but the chemical chaperones lost their beneficial effects at a higher concentration of 20 mmol/l (Fig. 3a, b). The exact mechanisms by which vitamins C and E restore pH at high glucose concentrations are not known. However, they are unlikely to be related to their antioxidant properties, as other antioxidants (edaravone and *N*-acetyl-l-cysteine) had no effect (data not shown). Nonetheless, both vitamin C and vitamin E protect muscle cells from mitochondrial damage, including membrane rupture, during exhaustive exercise, which is accompanied by lactate accumulation [38]. Therefore, the vitamins may maintain mitochondrial integrity and function during metabolic acidosis, thereby reducing anaerobic lactate production. Indeed, our data in Fig. 3d showing inhibition of lactate production support this speculation.

These results provide new insight into the potential use of ER chaperones as therapeutic agents in diabetic pregnancies. Additionally, vitamins C and E could be administered as health supplements in conjunction with both dietary and insulin control to mitigate the adverse effects of metabolic acidosis on the placenta, thereby improving fetal wellbeing. Furthermore, in a mouse model of diabetes, hyperglycaemia-induced ER stress mediated embryopathy can be ameliorated through administration of 4-PBA [39].

## Electronic supplementary material

Below is the link to the electronic supplementary material.

ESM (PDF 3.14 mb)

## Abbreviations

ATF6: Activating transcription factor 6
eIF2α: Eukaryotic initiation factor 2 subunit α
ER: Endoplasmic reticulum
GDM: Gestational diabetes mellitus
GRP: Glucose regulated protein
hPGH: Human placental growth hormone
hPL: Human placental lactogen
IRE1: Inositol-requiring enzyme 1
JNK: c-Jun N-terminal kinase
4-PBA: 4-Phenylbutyrate
PDI: Protein disulfide isomerase
PERK: Protein kinase RNA-like ER kinase
TUDCA: Tauroursodeoxycholic acid
UPR: Unfolded protein response
XBP-1: X-box binding protein 1
