# Supplementary material for: Placental endoplasmic reticulum stress in gestational diabetes: the potential for therapeutic intervention with chemical chaperones and antioxidants
Source: Diabetologia. 2016 Jul 12;59(10):2240–50. doi: 10.1007/s00125-016-4040-2 (PMC5016560; doi:10.1007/s00125-016-4040-2)
Supplement: Supplementary file 1 — (PDF 3.14 mb) [file 125_2016_4040_MOESM1_ESM.pdf]

ESM Methods

Antibodies

Antibodies against phospho-p38(Thr180/Tyr182), p38, phospho-JNK(Thr183/Tyr185), phospho-HSP27(Ser82) and HSP27 were from Cell Signalling Technology (NEB, Hitchin, UK). Antibody against HSP60 was from Abcam (Cambridge, UK), and HSP90 and HSP70 were from Enzo Life Science (Exeter, UK). All antibodies used according to manufacturer's instruction.

Generation of new line of BeWo cells, BeWo-NG and culturing conditions

Commercial DMEM/F12 medium contains 17.1 mmol/l glucose, and so represents a hyperglycemic environment. The BeWo-NG cells grew slowly at 5.5 mmol/l glucose concentration initially. After 10 passages, however, the proliferation rate was indistinguishable from that of the original BeWo line (data not shown), indicating the BeWo-NG cells had adapted to the more physiological glucose environment. Another 10 passages were performed to ensure full adaptation. All experiments were carried out using BeWo-NG cells after 20 passages. The cells were cultured in modified DMEM/F-12 medium containing 5.5 mmol/l glucose (Sigma-Aldrich, UK) supplemented with 10% heat-inactivated fetal bovine serum (Invitrogen, UK), penicillin (10,000 U/ml), and streptomycin (10,000 μg/ml) at 37°C in a 5% CO_2 atmosphere.

The BeWo-NG cells were induced to differentiate with 10 μM Forskolin A (Sigma-Aldrich, UK) in the presence 10% FBS for 24 h prior to experimentation. FSK A-treated BeWo-NG cells were rinsed with serum-free medium, and all experiments were carried out under serum-free conditions in the absence of FSK A.

Western Blot

Placental tissue lysates were prepared using MagNA Lyser Instrument (Roche, UK) with Lysing Martrix D (MP Biomedicals, UK) and lysis buffer containing 20 mmol/l Tris (pH 7.5), 150 mmol/l NaCl, 1 mmol/l, EDTA, 1 mmol/l EGTA, 1% Triton X-100, 2.5 mmol/l sodium pyrophosphate, 1 mmol/l β-glycerolphosphate, 1 mmol/l Na_3VO_4, and complete mini proteases inhibitor cocktail (Roche

Diagnostics, East Sussex, UK). Cell lysates after *in vitro* culture experiments were prepared using the same lysis buffer without homogenization. Protein concentration of the tissue lysate or cell lysate was determined by Bicinchoninic acid kit (Sigma-Aldrich, UK). Equivalent amounts of protein were resolved by SDSPAGE, blotted onto nitrocellulose (0.2 μm) and analyzed by enhanced chemiluminescence (ECL) (Amersham Biosciences, Bucks, UK) using Kodak X-OMAT Autoradiographic (AR) film (Sigma-Aldrich). β-actin or Ponceau S staining was used to normalize protein loading. Films were scanned using a flat-bed scanner (HP G4050) and intensities of the bands representing phospho- and total kinase forms were determined from two or three different exposures (within the linear detection range) using Image J analysis software (Freeware).

Immunocytochemistry

The syncytialization of BeWo cells was assessed by immunocytochemical staining with anti-Desmoplakin 1+2 antibody. Cells were fixed with acetone/methanol (1:1 v/v) at -20°C for 20 minutes. After extensively washed with PBS, the cells were permeabilized with saponin buffer containing 0.1% saponin (Sigma-Aldrich, UK) and 1% bovine serum albumin (Sigma-Aldrich, UK) in PBS for 30 minutes following by incubation with anti-human Desmoplakin 1+2 antibody (AbDSerotec, Oxford, UK) at 1 in 100 dilution in saponin buffer for overnight at 4°C following by a few hour at room temperature. After extensive washing with saponin buffer, cells were incubated with secondary anti-mouse antibody conjugated with Alexa Fluor 488 (Molecular Probes, San Diego, CA, USA) for 1 h at room temperature. Cells were then extensively washed in saponin buffer before briefly rinse in water and were mounted onto glass slides in an anti-fade solution containing DAPI nuclear dye (Vector Laboratories, Burlingame, CA, USA) and examined using a Zeiss AX10 fluorescence microscope (Carl Zeiss Ltd, Germany).

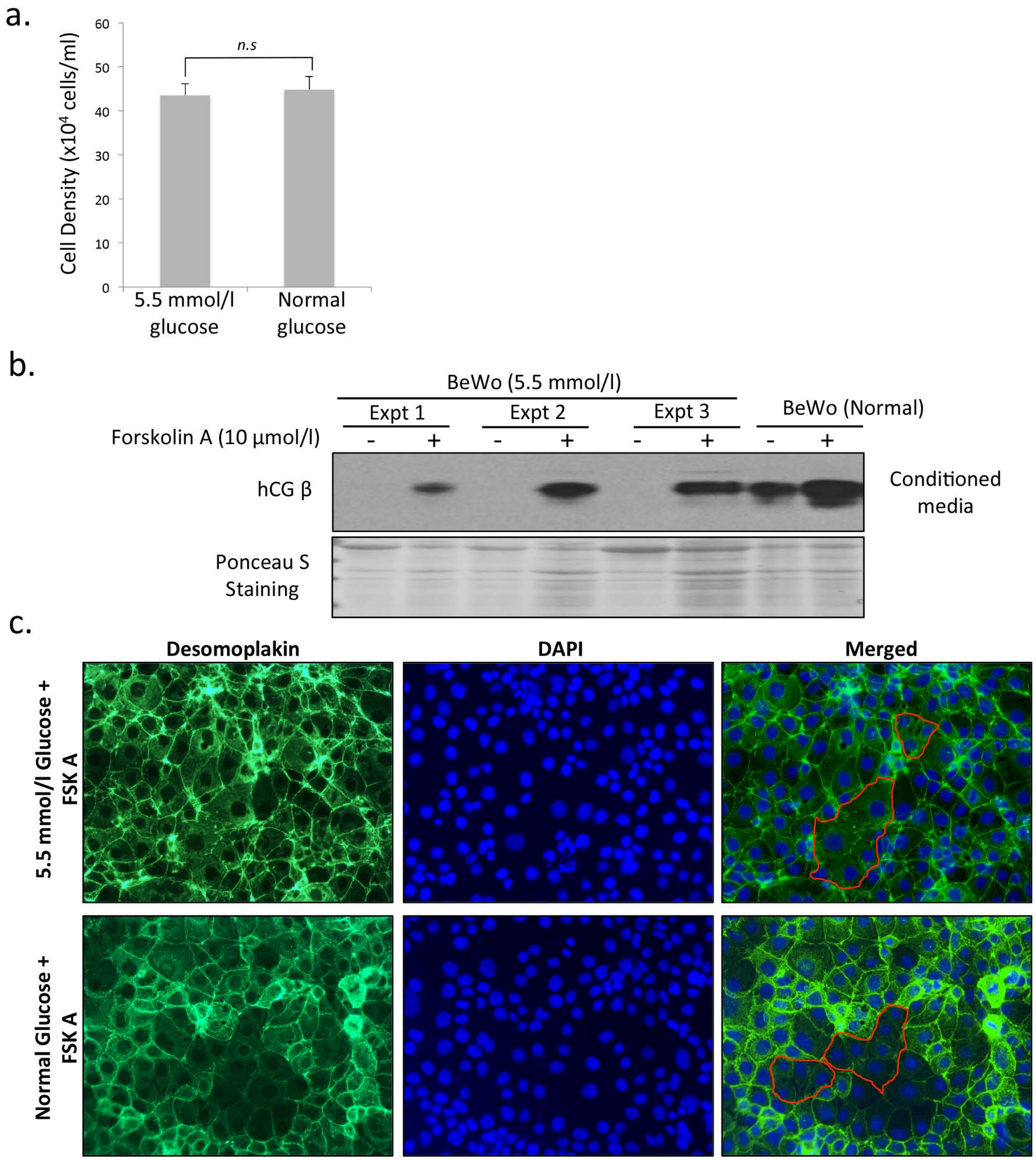

ESM Figure 1: *Validation of trophoblastic properties in newly derived BeWo-NG cells.* a) Cell proliferation rate in BeWo-NG cells is indistinguishable from parental BeWo cells. The number of cells was counted using a hematocytometer in BeWo-NG cells after 20 passages in parallel with parental BeWo cells with the same cell seeding density. Data are presented as mean ± SEM, n=3. b) BeWo-NG cells are able to produce hCG under the influence of forskolin A (FSK A). Confluent cells were treated with 10 µmol/l FSK A for 24 h and conditioned media were collected and concentrated. Equal volumes of conditioned media were subjected to Western blot analysis using hCGβ specific primary antibody. Ponceau S staining was used to show the amount of secreted proteins in each sample. c) BeWo-NG cells can undergo syncytialization after FSK A stimulation. The treatment was the same as the above. Cells were fixed in cold acetone/methanol and permeabilization using 0.1 % saponin in 1 % BSA. Cells were then stained with desmoplakin specific primary antibody and Alexa 488 conjugated secondary antibody before visualization using fluorescent microscopy. The nuclei of cells were counterstained with DAPI nuclear dye. Red lines were drawn around the boundary of syncytialized cells. Micrographs were taken under 200X magnification.

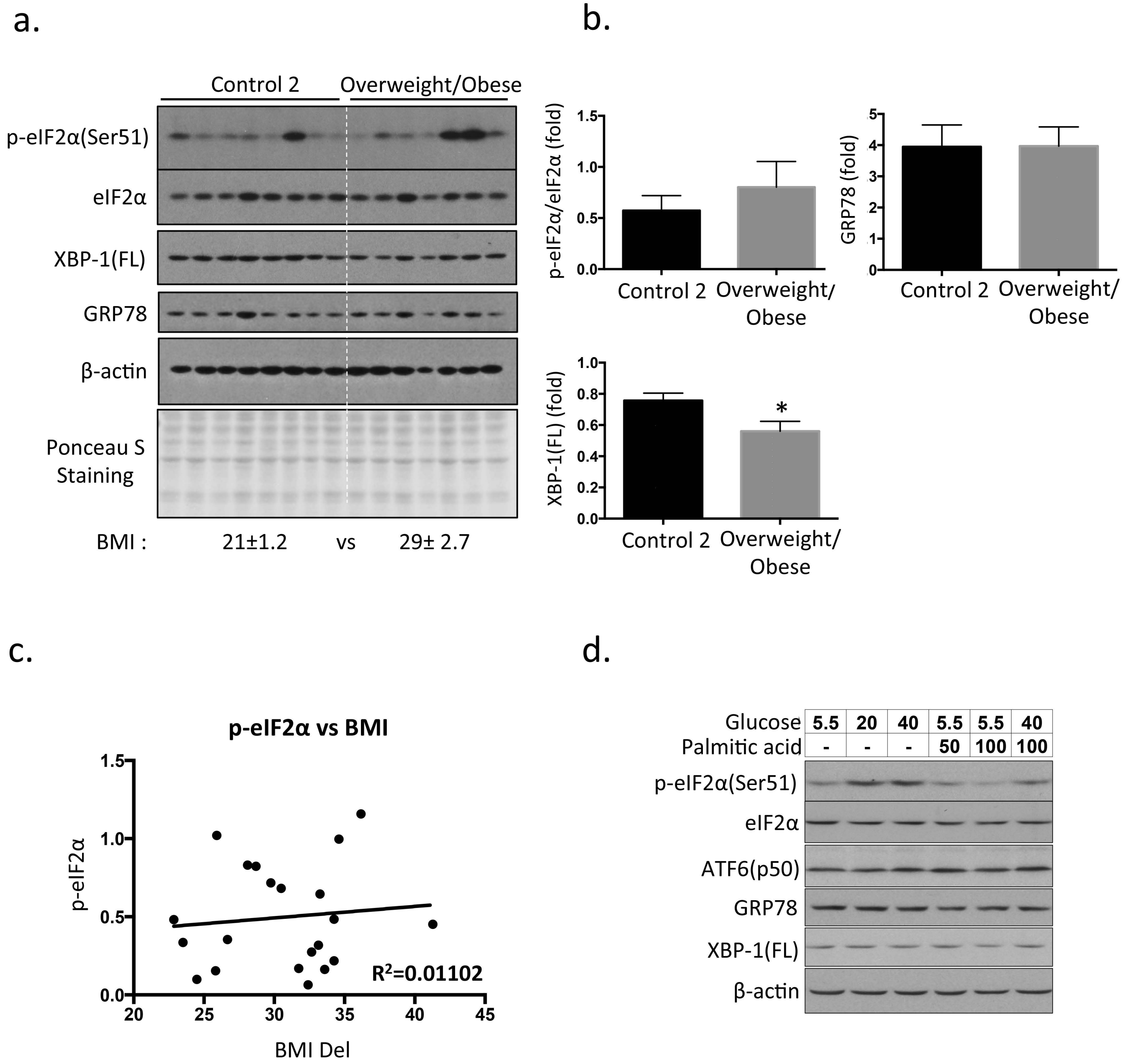

ESM Figure 2: *Obesity in GDM patients does not contribute to placental ER stress.* a) Placentas from overweight/obese women with normal pregnancies do not show ER stress. Both overweight/obese and gestational age matched control placentas were processed for examination of ER stress markers using Western blotting. The BMI in control and overweight/obese groups was 21 ± 0.2 and 29 ± 1 respectively. b) Quantitation of band intensity showed no difference in p-eIF2α and GRP78, but a significant reduction in XBP-1(FL) expression in the overweight/obese group. Data are presented as mean ± SEM, n = 7 or 8. * $p < 0.05$. c) The BMI of GDM patients was plotted against placental p-eIF2α level and a trend line was added. The correlation coefficient was calculated and presented as R^2. d) Addition of free fatty acid does not exacerbate ER stress in the presence or absence of high glucose challenge. BeWo-NG cells were treated for 24 h with 50 or 100 μmol/l of palmitic acid conjugated to BSA in the presence or absence of high glucose (20 or 40 mmol/l). Activation of ER stress markers was measured by Western blot using specific primary antibodies against corresponding proteins. β-actin was used as loading control.

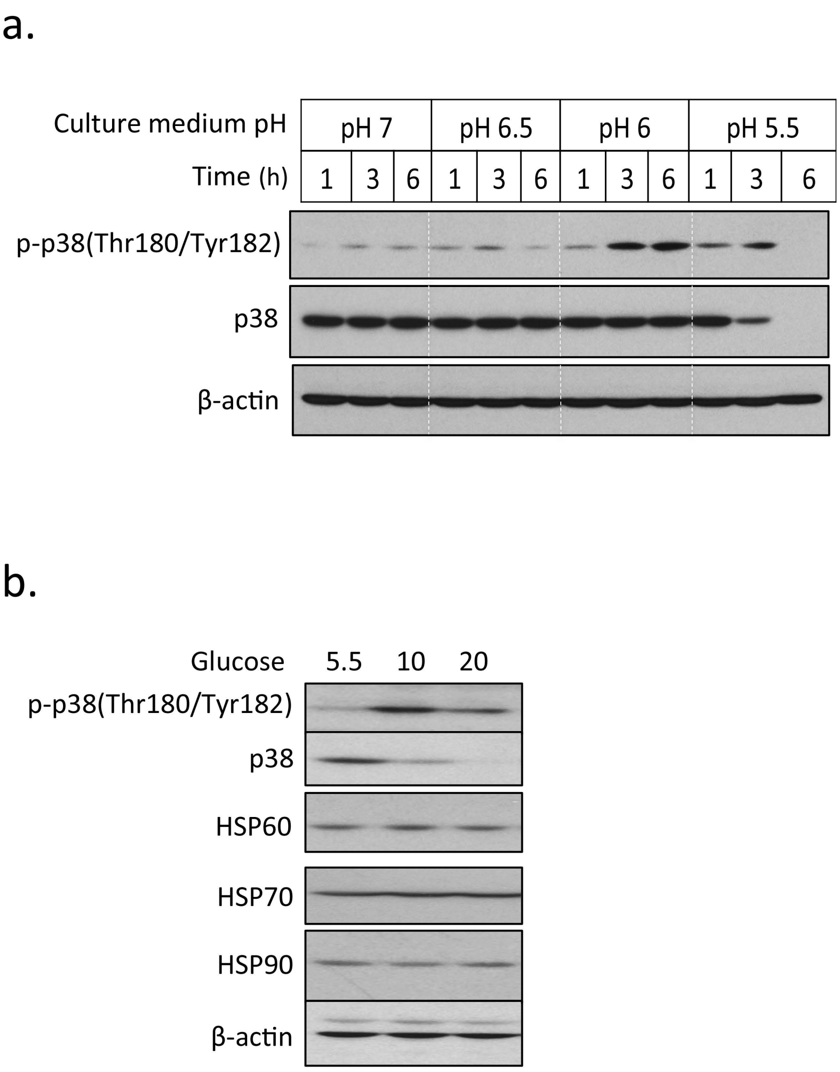

ESM Figure 3: *High glucose induces oxidative stress through metabolic acidosis.* a) Acidified culture media induced p38 kinase phosphorylation. The pH of the culture medium containing 5.5 mmol/l glucose was adjusted to 7, 6.5, 6 and 5.5 and the BeWo-NG cells were exposed to those culture media for 1, 3 or 6 h. The degree of oxidative stress was examined by Western blot. b) High glucose activated p38 kinase phosphorylation. BeWo-NG cells were treated with different concentrations of glucose under 2 ml for 24 h. The degree of oxidative stress was measured by Western blot with oxidative stress markers, p-p38, HSP90, HSP70 and HSP60.

a.

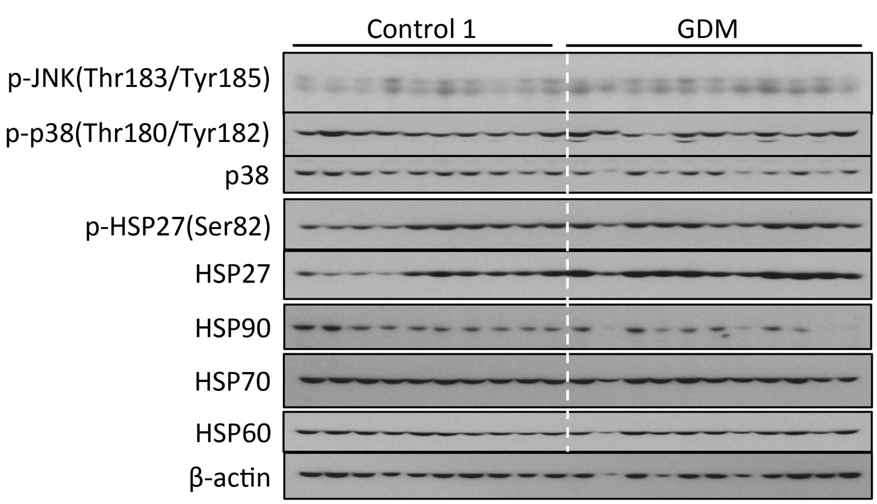

Control 1
GDM
p-JNK(Thr183/Tyr185)
p-p38(Thr180/Tyr182)
p38
p-HSP27(Ser82)
HSP27
HSP90
HSP70
HSP60
β-actin

b.

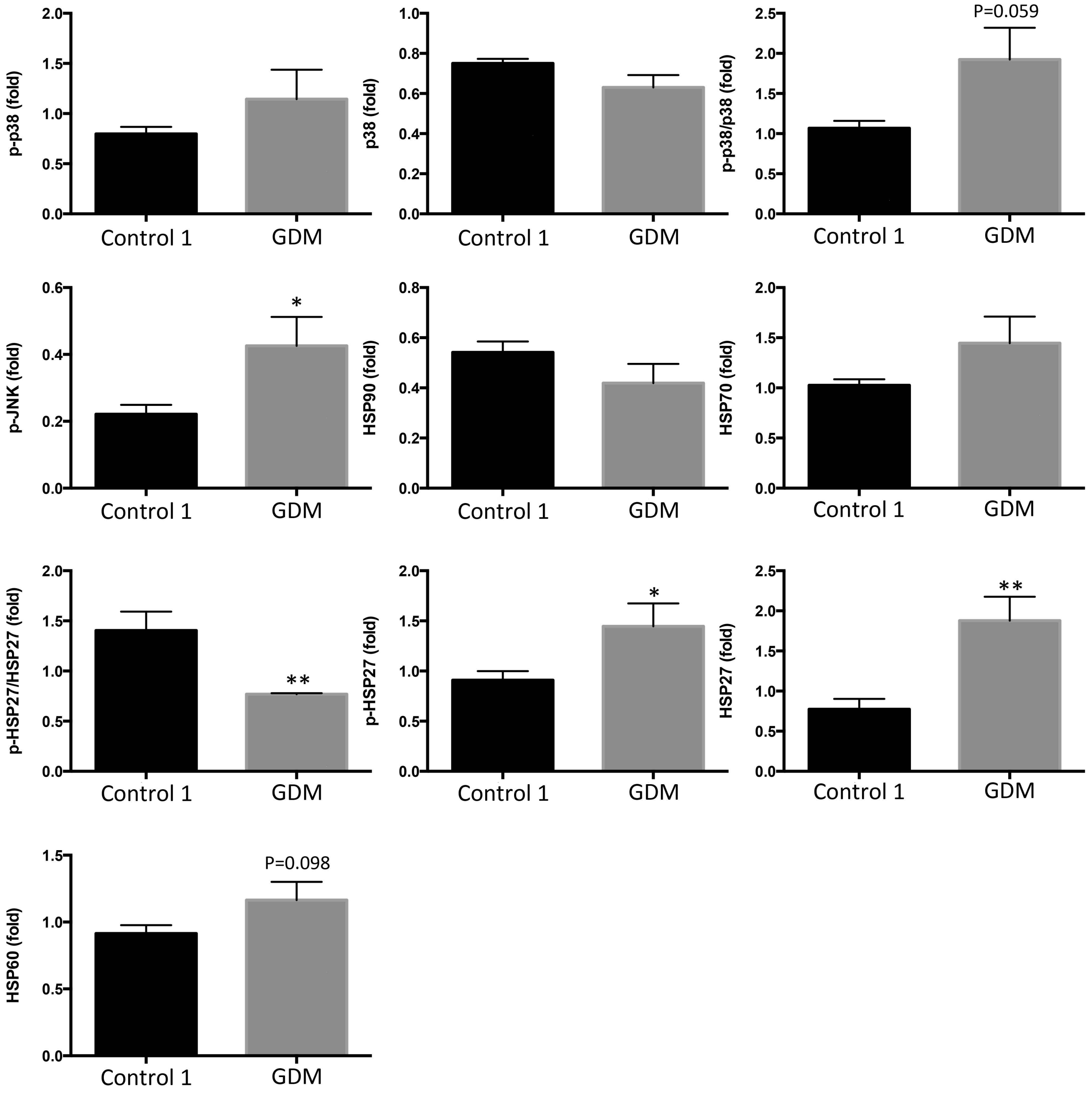

p-p38 (fold)
p38 (fold)
p-p38/p38 (fold)
P=0.059
p-JNK (fold)
*
HSP90 (fold)
HSP70 (fold)
p-HSP27/HSP27 (fold)
**
p-HSP27 (fold)
*
HSP27 (fold)
**
HSP60 (fold)
P=0.098
Control 1
GDM

c.

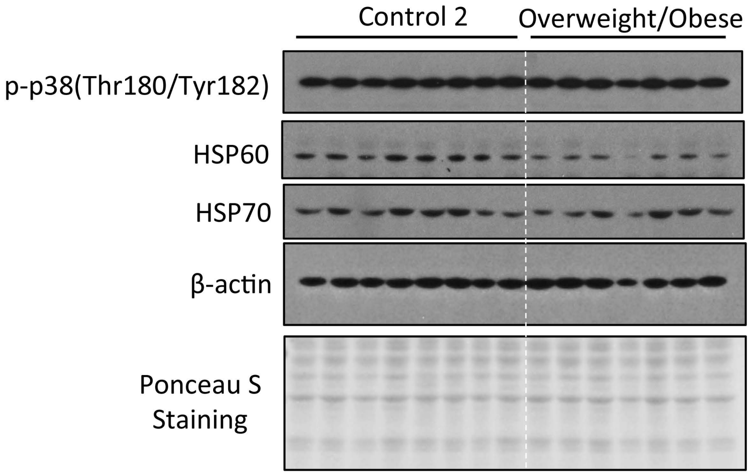

d.

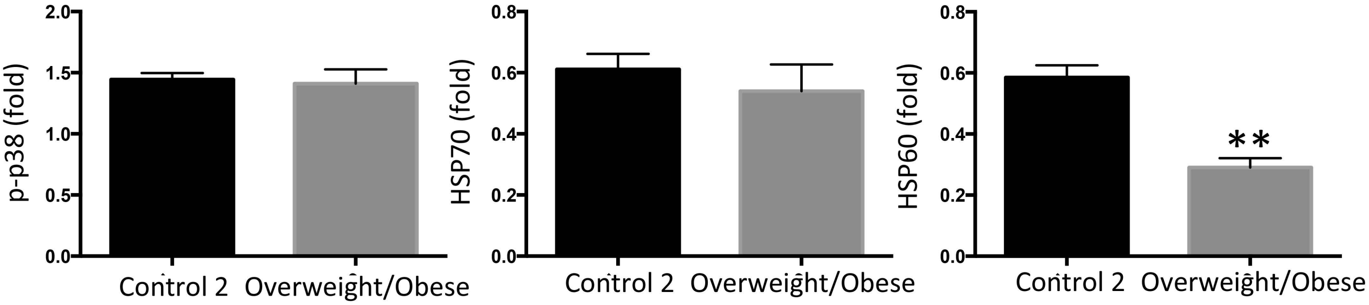

ESM Figure 4: *Increase of mild oxidative stress in GDM but not overweight/obese placentas.* a & c) Western blotting was used to measure oxidative stress markers in the GDM and overweight/obese placentas, including p-JNK, p-p38, HSP90, HSP70, HSP60, p-HSP27 and HSP27, with primary antibodies specific to corresponding proteins. β-actin was used as the loading control. b & d) Densitometry of band intensity is expressed in arbitrary units. Phosphorylation status is presented as the ratio between phosphorylated and total protein. Data are presented as mean ± SEM, n = 10 or 11. * & ** indicate $p \leq 0.05$ and $p \leq 0.01$ respectively.
